# 339. OPAT Outcomes in Patients with Immunocompromise

**DOI:** 10.1093/ofid/ofad500.410

**Published:** 2023-11-27

**Authors:** Monica V Mahoney, Kyleen Swords, Audrey Le Mahajan, Carolyn D Alonso, Jeffrey Larnard

**Affiliations:** Beth Israel Deaconess Medical Center, Boston, Massachusetts; Beth Israel Deaconess Medical Center, Boston, Massachusetts; Beth Israel Deaconess Medical Center, Boston, Massachusetts; Beth Israel Deaconess Medical Center, Boston, Massachusetts; Beth Israel Deaconess Medical Center, Boston, Massachusetts

## Abstract

**Background:**

Outpatient parenteral antimicrobial therapy (OPAT) allows patients to complete intravenous (IV) or high risk oral medications outside the acute inpatient setting. Data regarding OPAT in patients with immunocompromise (PWIC) are sparse. PWIC represent a vulnerable population due to underlying comorbidities. This study evaluated OPAT clinical outcomes in PWIC at an academic medical center.

**Methods:**

This was a retrospective chart review of adult PWIC enrolled in our OPAT program between 1/2015-3/2023. Immunocompromising conditions included HIV/AIDS, stem cell or solid organ transplant, hematologic malignancy, solid tumor, or receipt of immunosuppressing medications. All patients were under the care of an immunocompromised ID specialist. Data collected included patient demographics, infection information, and OPAT outcomes including completion rates, readmission rates, OPAT failure, and death. OPAT failure was defined as death, 30-day infection-related readmission or 30-day OPAT-related complication. This study was exempt by the Institutional Review Board.

**Results:**

50 patients were included. Demographics are in Table 1. Most (68%) patients completed OPAT therapy in the home, with 82% of courses being IV-only. Most (72%) patients received 1 antimicrobial agent, but 20% received 2 and 8% received 3 agents. The most common agents were daptomycin, ertapenem, and vancomycin.

Infection information is in Table 2. Bloodstream infections and osteomyelitis were the most common. Most (82%) patients had an organism identified: 51% were Gram positive, with *Staphylococci* the most prevalent. Cytomegalovirus was the reason for OPAT in 4 patients.

OPAT outcomes are in Table 3. OPAT failure occurred in 9 patients, including 1 death and 8 OPAT-related readmissions. Most (68%) patients completed the OPAT course within the specified time. Hospital readmissions were common: 46% had at least 1 readmission, though most readmissions were unrelated to OPAT. Vascular device issues and adverse drug reactions occurred in 12% and 20%, respectively.

**Figure:**
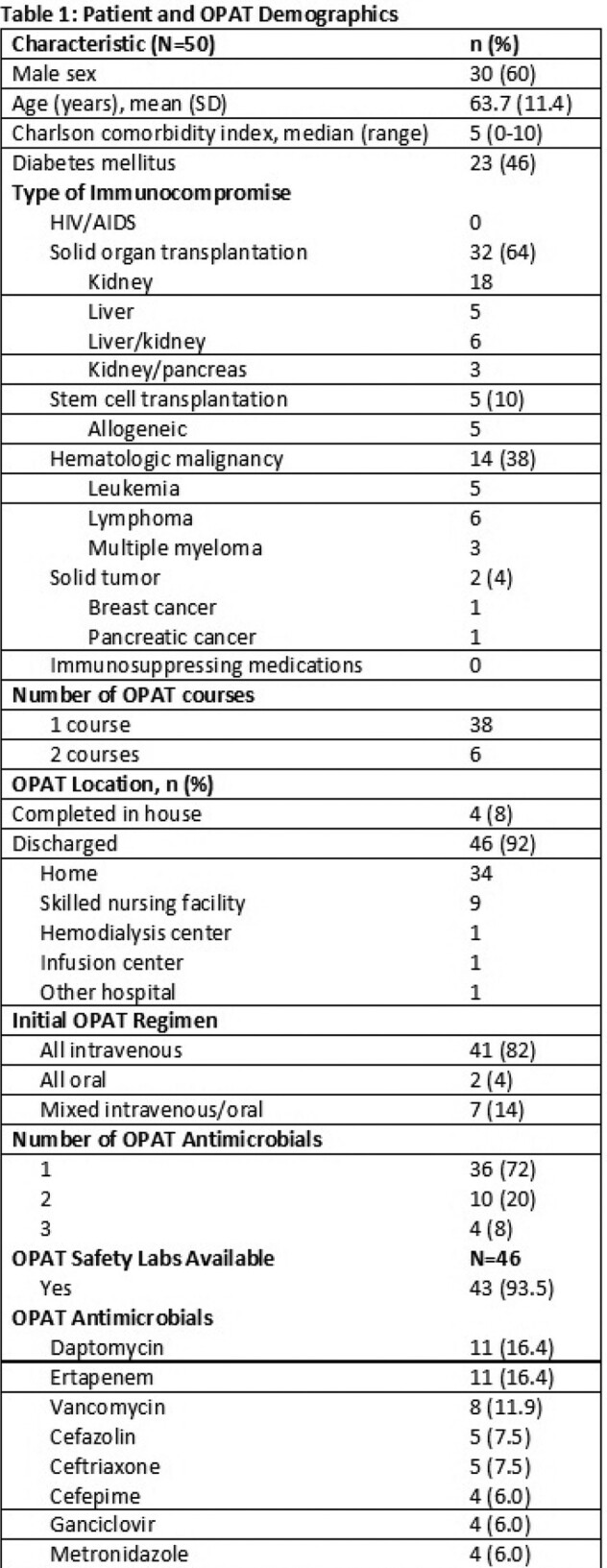

**Figure:**
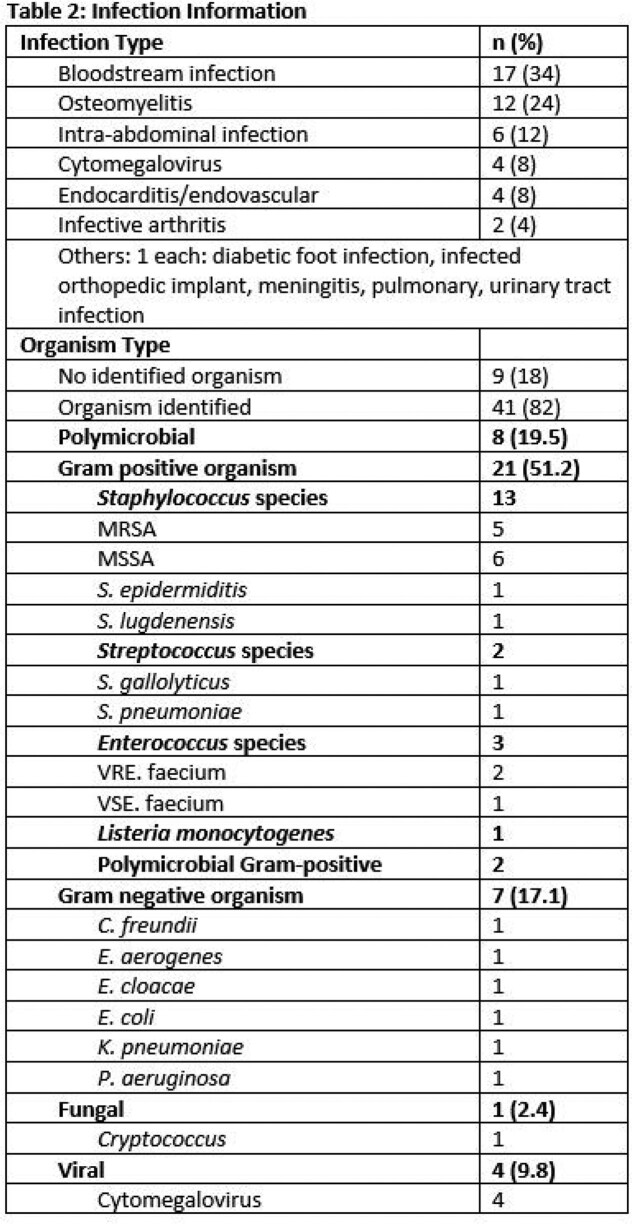

**Figure:**
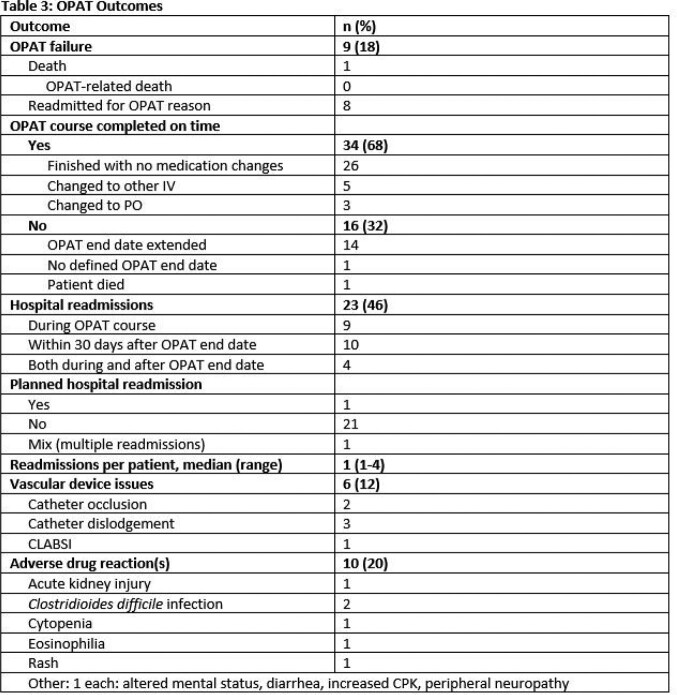

**Conclusion:**

OPAT can be safely performed in PWIC with completion and complication rates comparable to non-immunocompromised cohorts. We observed high hospital readmission rates (46%) though a minority of readmissions were due to OPAT related issues.

**Disclosures:**

**Monica V. Mahoney, PharmD, BCPS, BCIDP, FCCP, FIDSA, FIDP**, BD Biosciences: Advisor/Consultant|Cidara Therapeutics: Advisor/Consultant|GSK: Advisor/Consultant|Merck: Grant/Research Support|Open Forum Infectious Diseases: Board Member|Open Forum Infectious Diseases: Associate Editor|Pfizer: Advisor/Consultant **Carolyn D. Alonso, MD**, Academy for Continued Healthcare Learning: Honoraria|AiCuris: Advisor/Consultant|American Society of Healthcare Pharmacists: Honoraria|Cidara Therapeutics: Advisor/Consultant|Clinical Care Options: Honoraria|Merck: Advisor/Consultant|Merck: Grant/Research Support

